# Development and validation of a hypoxia-stemness-based prognostic signature in pancreatic adenocarcinoma

**DOI:** 10.3389/fphar.2022.939542

**Published:** 2022-07-21

**Authors:** Xiong Tian, Jing Zheng, Wanlan Mou, Guoguang Lu, Shuaishuai Chen, Juping Du, Yufen Zheng, Shiyong Chen, Bo Shen, Jun Li, Na Wang

**Affiliations:** ^1^ Department of Public Research Platform, Taizhou Hospital of Zhejiang Province Affiliated to Wenzhou Medical University, Linhai, China; ^2^ Department of Clinical Laboratory, Taizhou Hospital of Zhejiang Province Affiliated to Wenzhou Medical University, Linhai, China; ^3^ Department of Surgery, Taizhou Hospital of Zhejiang Province Affiliated to Wenzhou Medical University, Linhai, China

**Keywords:** pancreatic adenocarcinoma, hypoxia microenvironment, cancer stemness, mRNAsi, prognosis

## Abstract

**Background:** Pancreatic adenocarcinoma (PAAD) is one of the most aggressive and fatal gastrointestinal malignancies with high morbidity and mortality worldwide. Accumulating evidence has revealed the clinical significance of the interaction between the hypoxic microenvironment and cancer stemness in pancreatic cancer progression and therapies. This study aims to identify a hypoxia-stemness index-related gene signature for risk stratification and prognosis prediction in PAAD.

**Methods:** The mRNA expression-based stemness index (mRNAsi) data of PAAD samples from The Cancer Genome Atlas (TCGA) database were calculated based on the one-class logistic regression (OCLR) machine learning algorithm. Univariate Cox regression and LASSO regression analyses were then performed to establish a hypoxia-mRNAsi-related gene signature, and its prognostic performance was verified in both the TCGA-PAAD and GSE62452 corhorts by Kaplan-Meier and receiver operating characteristic (ROC) analyses. Additionally, we further validated the expression levels of signature genes using the TCGA, GTEx and HPA databases as well as qPCR experiments. Moreover, we constructed a prognostic nomogram incorporating the eight-gene signature and traditional clinical factors and analyzed the correlations of the risk score with immune infiltrates and immune checkpoint genes.

**Results:** The mRNAsi values of PAAD samples were significantly higher than those of normal samples (*p* < 0.001), and PAAD patients with high mRNAsi values exhibited worse overall survival (OS). A novel prognostic risk model was successfully constructed based on the eight-gene signature comprising JMJD6, NDST1, ENO3, LDHA, TES, ANKZF1, CITED, and SIAH2, which could accurately predict the 1-, 3-, and 5-year OS of PAAD patients in both the training and external validation datasets. Additionally, the eight-gene signature could distinguish PAAD samples from normal samples and stratify PAAD patients into low- and high-risk groups with distinct OS. The risk score was closely correlated with immune cell infiltration patterns and immune checkpoint molecules. Moreover, calibration analysis showed the excellent predictive ability of the nomogram incorporating the eight-gene signature and traditional clinical factors.

**Conclusion:** We developed a hypoxia-stemness-related prognostic signature that reliably predicts the OS of PAAD. Our findings may aid in the risk stratification and individual treatment of PAAD patients.

## Introduction

Pancreatic adenocarcinoma (PAAD) is one of the most aggressive and fatal gastrointestinal malignancies and has become the fourth leading cause of cancer-related deaths worldwide, severely threatening people’s lives (Rawla et al., 2019). Due to its occult and atypical clinical symptoms, PAAD is difficult to diagnose early. Most patients are diagnosed at a locally advanced stage or metastatic stage and are not eligible for surgical resection, which is the only curative therapy (Skelton et al., 2017). Despite new advances in comprehensive treatment, targeted molecular therapy and immunotherapy, PAAD patients still experience a dismal prognosis with an average 5-year survival rate of less than 5% due to late detection, drug resistance and postoperative metastasis and recurrence (Siegel et al., 2019). For patients with an increased risk of poor outcomes, individualized systemic treatments may help prolong survival and improve quality of life. Therefore, there is still an urgent need to develop an effective predictive model to accurately evaluate the survival outcomes of PAAD patients and provide support for clinical decision making.

Increasing evidence has shown that tumor cell proliferation and growth are highly dependent on the existence of a functional subpopulation of cancer stem cells (CSCs) that play critical roles in tumor metastasis, recurrence and chemoresistance (Tanase et al., 2014; Alferez et al., 2018). Prior research suggests that CSCs promote the development and progression of pancreatic cancer, and high expression levels of CSC markers such as CD44 and CD133 are strongly correlated with neoplasm recurrence and poor prognosis in PAAD (Stoica et al., 2020). Intratumoral hypoxia is a prominent feature of the pancreatic tumor microenvironment facilitating tumor metastasis and invasion and is closely associated with disease progression and poor survival in patients with pancreatic cancer (Erkan et al., 2016). Recent work has established that hypoxia contributes to the induction and maintenance of CSC stemness by upregulating the expression of hypoxia-inducible factors (HIFs), while blockade of HIF-1 activity decreases CSC marker expression and weakens the CSC population (Hao, 2015; Tong et al., 2018; Zeng et al., 2019; Mortezaee, 2021). In addition, Zhang et al. (2018) proposed that hypoxia might synergistically potentiate chemotherapy resistance through stemness induction, highlighting the clinical significance of hypoxia-CSC interactions in the PAAD microenvironment.

Recently, Malta et al. (2018) derived a novel stemness index (mRNAsi) that could reflect the stemness features of cancer samples based on the theory of CSCs using a one-class logistic regression (OCLR) machine learning algorithm. mRNAsi was found to be closely correlated with overall survival (OS) in pancreatic cancer, and patients with high mRNAsi values showed poor prognosis (Tang et al., 2021). In addition, Huang et al. (2021) established an mRNAsi-related prognostic model to successfully predict patient survival in pancreatic cancer. Moreover, multigene prognostic signatures based on the hypoxic microenvironment have been verified to be prognostic markers for pancreatic cancer and could be applied for risk stratification and clinical treatment (Abou Khouzam et al., 2021; Chen et al., 2021; Ding et al., 2021). Hence, targeting the hypoxic microenvironment and establishing reliable prognostic signatures based on the combination of hypoxia and cancer stemness may provide new perspectives for personalized disease management and therapeutic strategies in PAAD.

In this study, we aimed to explore the prognostic value of a hypoxia-stemness-based gene signature in PAAD. Based on the mRNA profile data of PAAD patients from The Cancer Genome Atlas (TCGA) database, we analyzed the correlation between mRNAsi and prognosis. A total of 45 hypoxia-stemness-related genes (HSRGs) with prognostic value were then identified, and functional enrichment analysis was performed to reveal the potential functions of these genes in the pathogenesis and progression of PAAD. A novel prognostic risk model including eight genes (JMJD6, NDST1, ENO3, LDHA, TES, ANKZF1, CITED and SIAH2) was then established, and its predictive performance was verified in both the training dataset and external validation dataset. Besides, we further validated the expression levels of signature genes using the TCGA, GTEx and HPA databases as well as qPCR analysis. Finally, we constructed a nomogram for prognostic prediction in PAAD and analyzed the correlations among the risk score, immune infiltrates and immune checkpoint genes. Overall, our prognostic gene signature and nomogram might be useful tools for the risk stratification and prognosis prediction of PAAD patients.

## Materials

### Data collection and processing

The RNA-sequencing (RNA-seq) data and clinical information of 180 PAAD samples were downloaded from the TCGA database. The expression profile and clinical data of 130 PAAD samples in the GSE62452 microarray dataset were downloaded from the Gene Expression Omnibus (GEO) database. After the removal of the samples without survival information, a total of 162 PAAD tumor samples in the TCGA database were included in the further analysis as a training cohort, and a total of 65 PAAD tumor samples in GSE62452 were enrolled as an external validation cohort. Since the data used in the present study were obtained from the open database for free, the approval of the Ethics Committee was not needed.

### mRNAsi in PAAD and its prognostic value

Through the “gelnet” R package (Version 1.2.1, https://CRAN.R-project.org/package=gelnet), the mRNAsi score of PAAD tumor samples and control samples in the TCGA dataset was calculated by using the OCLR machine learning algorithm (Wei et al., 2021). A significant difference in mRNAsi values between PAAD tumor tissues and normal samples was defined using the intergroup *t* test, and all PAAD tumor samples were then divided into two groups, namely, the low-mRNAsi and high-mRNAsi groups, according to the median value of mRNAsi. In addition, the association between mRNAsi and the OS of PAAD patients in the two groups was analyzed using the Kaplan-Meier curve method with the “survival” R package.

### Identification of HSRGs

The hypoxia-related genes were downloaded from the hallmark gene set in the GSEA database (http://www.gseamsigdb.org/gsea/downloads.jsp), and their expression levels were extracted from TCGA PAAD samples. The Pearson correlation coefficient (PCC) between the expression level and mRNAsi value of each hypoxia gene was then calculated using the Cor function in R software (http://77.66.12.57/R-help/cor.test.html). Genes with thresholds of |PCC| > 0.4 and *p* < 0.05 were determined to be significantly associated with mRNAsi.

### PPI network construction and functional enrichment analysis

The STRING database (http://string-db.org/) was utilized to analyze the functional interactions among the encoded proteins of HSRGs, and those interaction pairs with an interaction score >0.4 were selected to construct a protein-protein interaction (PPI) network, which was then visually presented through Cytoscape (version 3.6.1, http://www. cytoscape.org/). In addition, we performed enrichment analysis of the Gene Ontology (GO) and Kyoto Encyclopedia of Genes and Genomes (KEGG) pathways to reveal the underlying biological functions and signaling pathways of HSRGs. *p* < 0.05 and FDR <0.05 were considered significantly different.

### Construction and validation of a prognostic gene signature model

Through the “survival” R package, we conducted univariate Cox regression analysis to identify HSRGs significantly associated with OS (*p* < 0.05) and ultimately screened out survival-related HSRGs. The least absolute shrinkage and selection operator (LASSO) regression algorithm in the “lars” R package was subsequently applied to acquire the optimal OS-related HSRGs. Then, the risk model was constructed based on the expression levels and LASSO coefficients of the eight-gene signature. Finally, the risk score of each sample was calculated as follows:

$$\mathrm{Risk}\;Score=\sum \mathrm{Coe}f_{\mathrm{genes}}\mathrm{\times Ex}p_{\mathrm{genes}}$$

In the formula, Coef_genes_ represents the LASSO coefficient of the target gene, and Exp_genes_ represents the gene expression level.

The risk scores of PAAD patients in TCGA training cohort and GSE62452 validation cohort were calculated, and all patients were then classified into high-risk and low-risk group according to the median value of risk score served as the cutoff value. Kaplan-Meier analysis was performed to analyze the correlation between the risk score and OS. Receiver operating characteristic (ROC) curves were then used to assess the predictive accuracy of the risk model. To further verify the predictive performance of the prognostic risk model, we also performed survival analysis and ROC analysis in the GSE62452 external validation dataset.

### Exploration of the mRNA and protein expression levels of the eight signature genes

The expression levels of JMJD6, NDST1, ENO3, LDHA, TES, ANKZF1, CITED, and SIAH2 were compared between PAAD tumor tissues and normal tissues using the TCGA and Genotype-Tissue Expression (GTEx) databases, and the Human Protein Atlas (HPA) database (https://www.proteinatlas.org/) was used to investigate the protein expression levels.

### Cell culture and qRT-PCR analysis

The human pancreatic cancer cell lines (BxPC-3, SW1990 and PANC-1) and the normal human pancreatic ductal epithelial cell line (HPNE) were purchased from Cell Bank of Chinese Academy of Sciences (Shanghai, China) and Mingzhou Biotechnology Co., LTD. (Ningbo, China). BxPC-3 and PANC-1 cells were cultured in RPMI 1640 medium (HyClone, United States) with 10% fetal bovine serum (FBS) and 1% penicillin-streptomycin at a humidified incubator at 37°C with 5% CO_2_, while SW1990 cells was 90% L-15 medium with 10% fetal bovine serum (FBS) at a humidified incubator at 37°C with 100% air. Meanwhile, HPNE cells were cultured in 70.5% glycoprival DMEM medium with 5% fetal bovine serum (FBS), 23.5% M3 medium, 10 ng/ml human recombinant EGF, 750 mg/ml puromycin, 2.5 g/L D-glucose and penicillin-streptomycin at a humidified incubator at 37°C with 95% air and 5% CO_2_.

Total RNA from the normal human pancreatic ductal epithelial cells and human pancreatic cancer cells was extracted using TRIzol reagent (Thermo Fisher Scientific, MA, United States), and 1 μg of total RNA for reverse transcription was prepared using the PrimeScript RT reagent Kit with gDNA Eraser (Toyobo, Japan). Reverse transcription quantitative PCR was performed under the following cycling conditions: 95°C for 3 min, followed by 40 cycles of 95°C for 10 s and 60°C for 30 s. The relative mRNA expression levels of JMJD6, NDST1, ENO3, LDHA, TES, ANKZF1, CITED, and SIAH2 were normalized to GAPDH expression, and calculated by the 2^−ΔΔCt^ method. The primer sequences are presented in Supplementary Table S1.

### Establishment and assessment of a prognostic nomogram

The clinical characteristics of PAAD patients, including age, sex, tumor size, lymph node metastasis, distant metastasis, tumor stage, chronic pancreatitis history, diabetes history, alcohol history, tobacco history, radiotherapy and recurrence, were extracted from the TCGA dataset, and Fisher’s exact test was used to compare the differences between the low- and high-risk groups. Subsequently, univariate and multivariate Cox regression analyses were conducted to identify independent prognostic factors (log rank *p* < 0.05). Through the “rms” R package, we established a nomogram integrating the risk score, age and targeted molecular therapy to predict the 1-, 3- and 5-year OS rates of PAAD patients. A calibration curve was plotted to evaluate the agreement between the predicted and observed OS probabilities. The concordance index (C-index) was calculated with the “survcomp” R package to evaluate the predictive accuracy of the nomogram, and a C-index >0.70 indicated a good predictive model.

### Gene set variation analysis

To further investigate the variation in biological pathways in PAAD patients between the low- and high-risk groups in the training dataset, GSVA enrichment analysis was carried out using the “GSVA” R package. The KEGG and genetic data were downloaded from the GSEA database for GSVA. Adjusted *p* < 0.05 was considered statistically significant.

### Correlation between the risk score and the immune microenvironment

Next, we explored the association between the risk score and the tumor immune microenvironment (TIME), including immune cell infiltration patterns and immune checkpoint molecules. The immune cell type in tumors was classified by utilizing the CIBERSORT method (Chen et al., 2018), and we obtained a total of 22 kinds of immune infiltrating cells, including T cells, B cells, dendritic cells, monocytes, neutrophils, natural killer (NK) cells, eosinophils and mast cells. The immune checkpoint genes included in this study were PDCD1/PD-1, PD-L1/CD274, CTLA4, CD278, CD366, LAG3/CD223, CD73, CD47, BTLA, TIGIT, MYD1, TNFRSF4, TNFRSF9, and VTCN1.

### Statistical analysis

Statistical analyses were conducted using R software (version 3.6.1) and GraphPad Prism 8 software (GraphPad Software, CA, United States). All statistical tests with *p* < 0.05 (two-sided) were considered statistically significant.

## Results

### mRNAsi in PAAD and survival analysis

**FIGURE 1:**
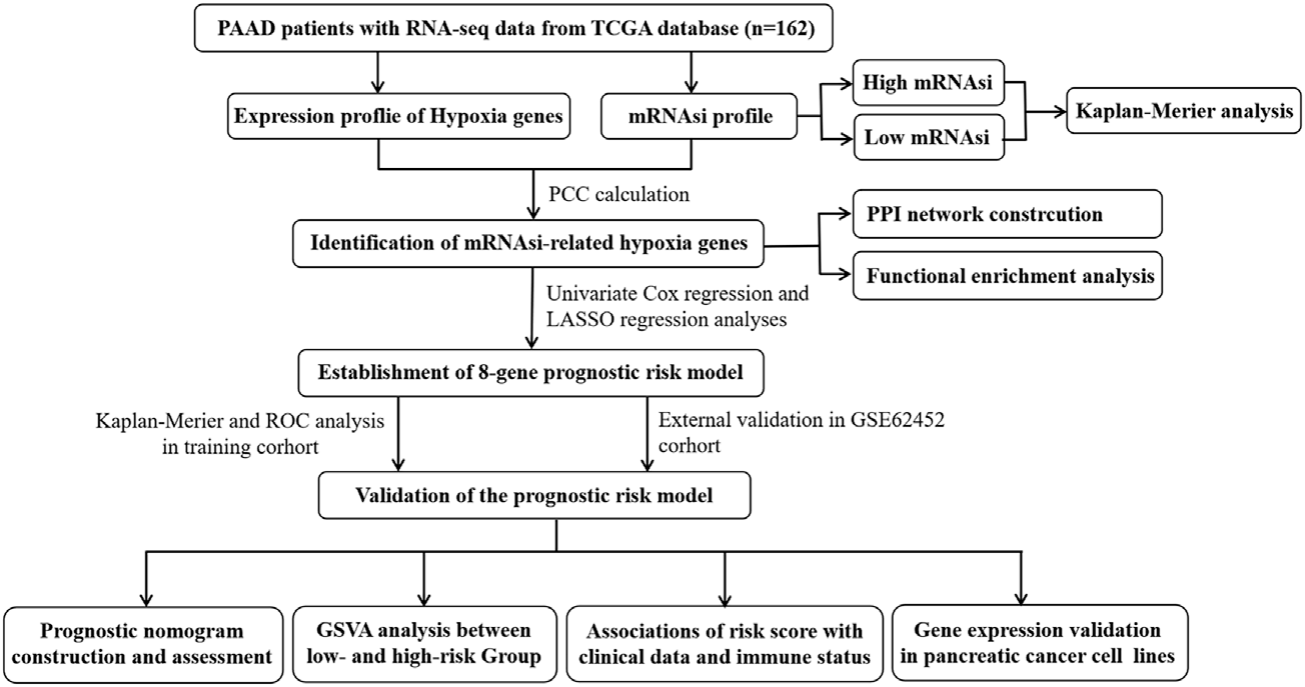
Overall flow chart of our current work.

**FIGURE 2:**
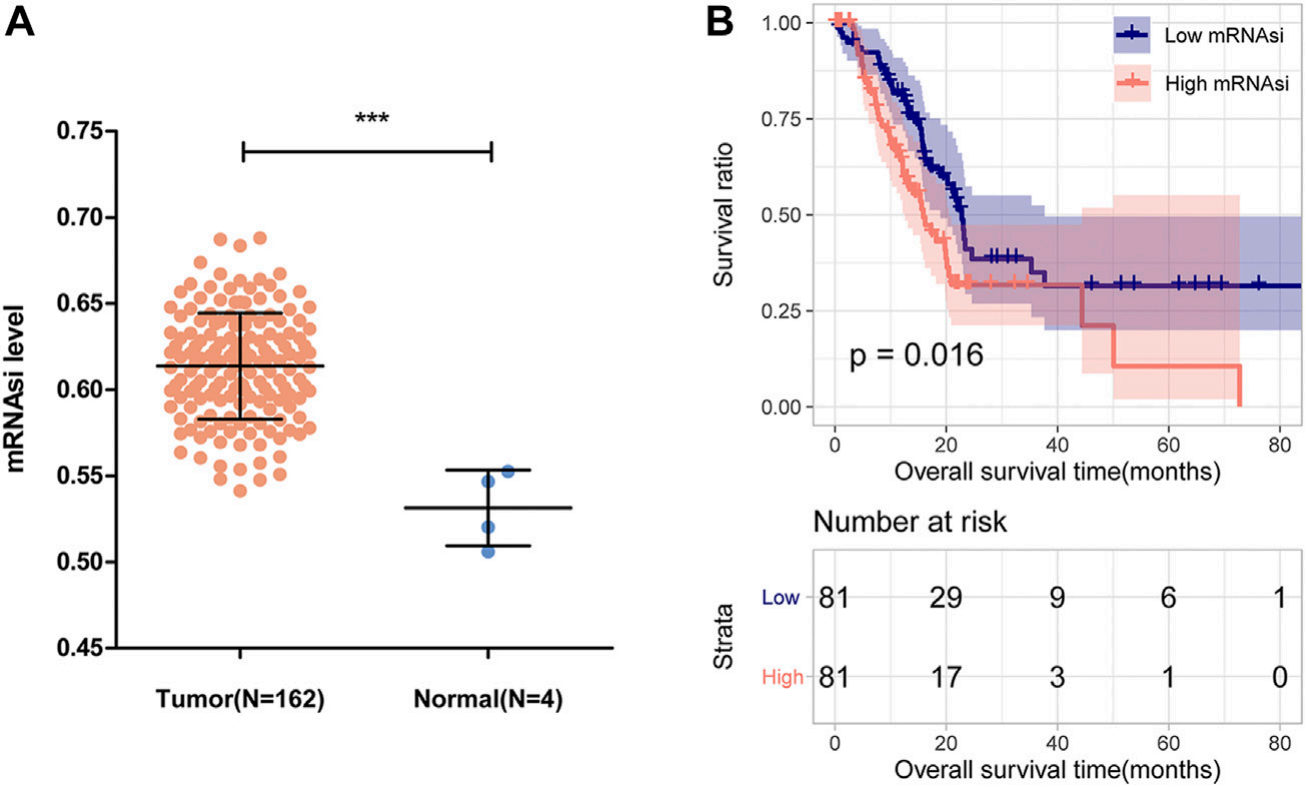
mRNAsi and its prognostic value in PAAD. **(A)** Comparison of mRNAsi between PAAD tumor tissues and normal tissues in the TCGA dataset. **(B)** Kaplan-Meier curve analysis of patients in the low- and high-mRNAsi groups.

This study was performed according to the flow chart presented in Figure 1. The mRNAsi of each PAAD sample was calculated *via* the OCLR algorithm based on the mRNA profile data downloaded from the TCGA database. As shown in Figure 2A, the mRNAsi in PAAD tumor tissues was remarkably higher compared with that in normal tissues. All PAAD patients were then divided into low- and high-mRNAsi groups based on the median value of mRNAsi, and survival analysis revealed that PAAD patients with high mRNAsi values had a worse prognosis than those with low mRNAsi values (Figure 2B).

### Identification of HSRGs

We downloaded the hallmark gene set from the GSEA database and obtained a total of 200 hypoxia-related genes, and a total of 108 HSRGs were identified according to the threshold criteria |PCC| > 0.4 and *p* < 0.05 (Supplementary Table S2). These genes were then arranged in order of PCC from lowest to highest, and correlation analysis of these genes was performed to determine the association between gene expression and mRNAsi. The top 3 hypoxia genes exhibiting negative and positive correlations with mRNAsi are represented in Supplementary Figure S1.

### PPI network construction and functional enrichment analysis

**FIGURE 3:**
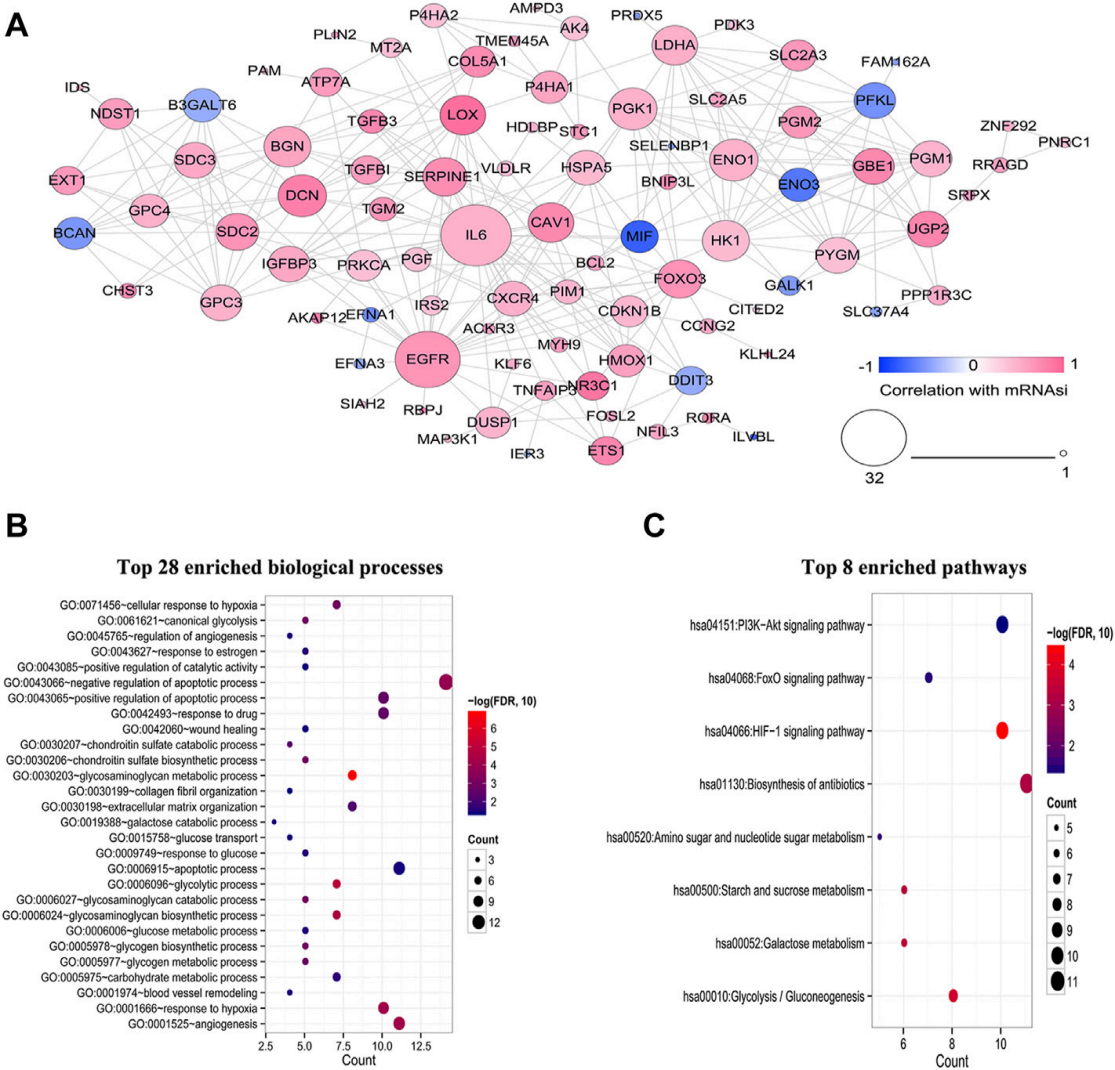
Construction of a PPI network and functional enrichment analysis of HSRGs. **(A)** A PPI network with 29 nodes and 297 edges was constructed to evaluate protein interactions. **(B)** Top 28 enriched biological processes. **(C)** Top 8 enriched KEGG pathways.

At the protein level, the interactions among 108 HSRGs products were evaluated using STRING, and a PPI network consisting of 92 nodes and 297 edges was constructed (Figure 3A). In addition, GO and KEGG pathway enrichment analyses were carried out to reveal the biological functions of these genes and participating pathways. The results showed that they were significantly enriched in biological processes, including glycosaminoglycan metabolic process, glycolytic process, angiogenesis, response to hypoxia and negative regulation apoptotic process (Figure 3B). KEGG pathway analysis suggested that these genes were principally involved in the HIF-1 signaling pathway and glycolysis/gluconeogenesis (Figure 3C).

### Construction of the eight-gene prognostic signature in PAAD

A total of 45 HSRGs strongly correlated with OS were identified using univariate Cox regression analysis (Supplementary Table S3). A novel prognostic signature composed of eight genes, including Jumonji domain containing 6 (IMJD6), N-deacetylase-N-sulfotransferase-1 (NDST1), enolase 3 (ENO3), lactate dehydrogenase A (LDHA), testin LIM domain protein (TES), ankyrin repeat and zinc finger peptidyl tRNA hydrolase 1 (ANZF1), CBP/p300-interacting transactivator with glutamic acid/aspartic acid-rich carboxyl-terminal domain 2 (CITED2) and seven in Absentia Homolog 2 (SIAH2), was successfully established by LASSO-penalized regression analysis (Supplementary Figure S2). Subsequently, the risk score of PAAD patients in the training cohort was calculated according to the following formula: Risk score = [(−0.003993857) × Expression value of JMJD6] + [(−0.010590801) × Expression value of NDST1] + [(−0.090216539) × Expression value of ENO3]+[0.00552938 × Expression value of LDHA] + [0.085804528 × Expression value of TES] + [(−0.021876003) × Expression value of ANKZF1] + [(−0.040066157) × Expression value of CITED2] + [0.114809344 × Expression value of SIAH2].

### Internal and external validation of the predictive performance of the eight-gene signature

**FIGURE 4:**
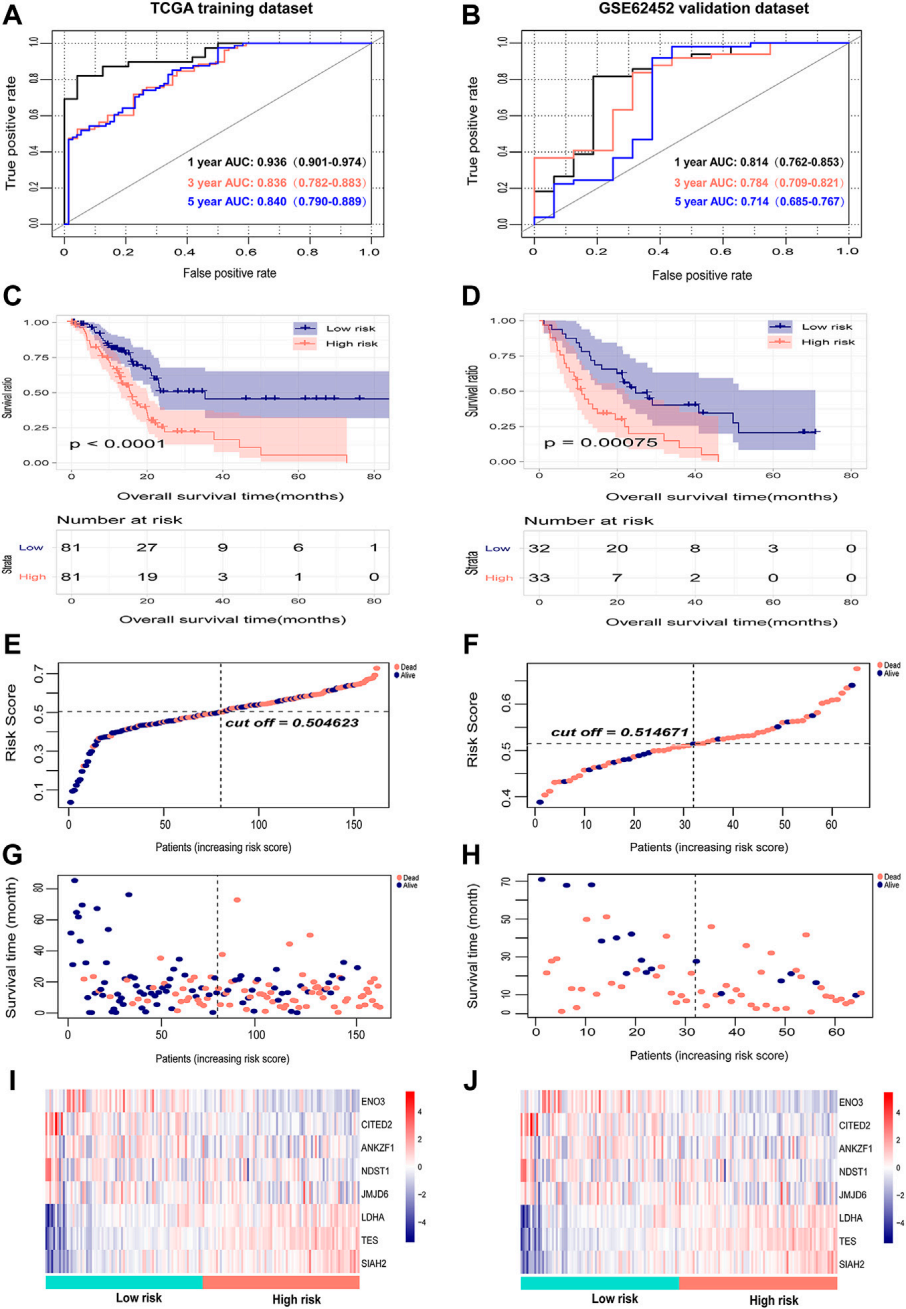
Internal evaluation and external validation of the prognostic performance of the eight-gene signature. **(A,B)** Time-dependent ROC analysis of the eight-gene signature for survival prediction in the TCGA training cohort and GSE62452 testing cohort. **(C,D)** Kaplan-Meier analysis of the correlation between the risk score and the OS of PAAD patients. **(E,F)** The distribution of the eight-gene risk scores of each PAAD patient. **(G,H)** Survival status of PAAD patients ranked by risk score. **(I,J)** The mRNA expression heatmap of the eight genes in the low- and high-risk groups. Red represents upregulation, and blue represents downregulation.

The risk scores of PAAD patients in the TCGA training dataset and the GSE62452 external validation dataset were calculated, and the optimal cutoff values of the risk score in the TCGA and GSE62452 datasets were 0.5 and 0.51, respectively (Figures 4E,F). PAAD patents in both datasets were then divided into high-risk and low-risk groups according to the median value. The survival time, survival status, and gene expression of eight genes in each PAAD patient in both datasets are shown in Figures 4E–J. Kaplan-Meier survival analysis revealed a notable correlation between high risk scores and the dismal prognosis of PAAD patients in the TCGA dataset (Figure 4A). The prognostic value of the eight-gene signature was assessed by utilizing time-dependent ROC, and the area under the curve (AUC) values for 1-, 3-, and 5-year OS were 0.936 (95% CI: 0.901–0.974), 0.836 (95% CI: 0.782–0.883) and 0.840 (95% CI: 0.790–0.889), respectively (Figure 4C), demonstrating the good predictive efficacy of the eight-gene signature in predicting the OS of PAAD patients. Corresponding with the results of the TCGA dataset, the survival outcomes of patients in the high-risk group were significantly worse than those of patients in the low-risk group in the GSE62452 dataset (Figure 4B). Time-dependent ROC analysis suggested that the AUCs for 1-, 3-, and 5-year OS were 0.814 (95% CI: 0.762–0.853), 0.784 (95% CI: 0.709–0.821), and 0.714 (95% CI: 0.685–0.767), respectively (Figure 4D), which confirmed the stability of the eight-gene signature in survival prediction for PAAD.

### Validation of the expression and alteration of the eight genes in PAAD tissues

**FIGURE 5:**
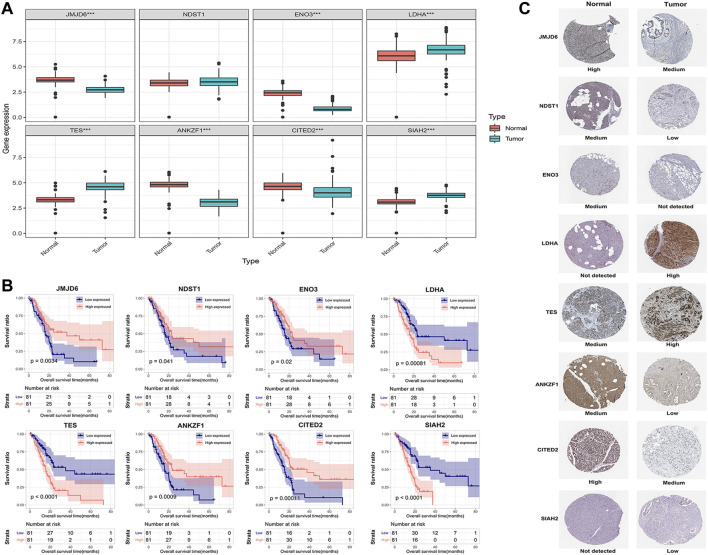
Validation of the expression of the eight signature genes in PAAD. **(A)** The mRNA expression profile of the eight genes in tumor tissues from the TCGA database and normal pancreatic tissues from the TCGA and GTEx databases. **(B)** Kaplan-Meier curve of the association between the mRNA expression levels of the eight genes and the OS of PAAD patients. **(C)** The protein expression of the eight genes in pancreatic tumor tissues and normal tissues. The data were obtained from the HPA database. ENO3 was not found in the database.

**FIGURE 6:**
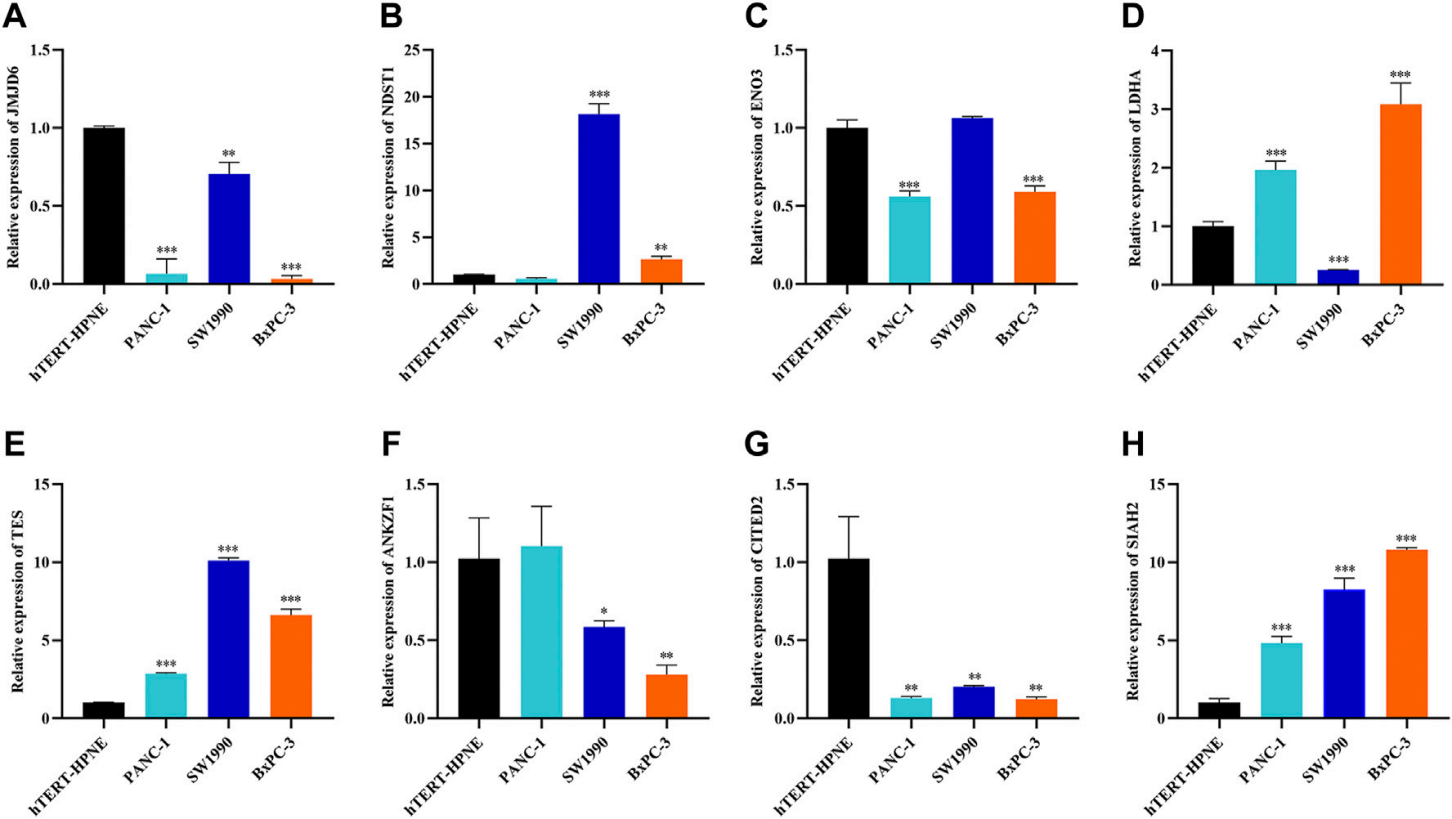
Further verification of the mRNA expression levels of seven genes in human pancreatic cancer cell lines and human pancreatic ductal epithelial cell line by RT-qPCR analysis. *p < 0.05, **p < 0.01, ***p < 0.001.

To explore the clinical significance of the eight genes in the model, their mRNA expression levels were validated using the TCGA and GTEx databases. As shown in Figure 5A, the mRNA expression levels of LDHA, TES and SIAH2 were obviously increased in PAAD tissues, while those of JMJD6, ANKZF1, ENO3 and CITED2 were significantly decreased in PAAD. HPA analysis showed that the protein levels of LDHA and TES were highly upregulated in cancer tissues compared to normal pancreatic tissue, while those of NDST1, ANKZF1, and SIAH2 were significantly decreased in cancer tissues. However, ENO3 was not detected in PAAD tumor tissues in the HPA database (Figure 5C). In addition, the Kaplan-Meier curve method was used to explore the effect of these eight genes on PAAD prognosis. We found that PAAD patients with overexpression of JMJD6, NDST1, ENO3, ANKZF1, and CITED2 had a relatively favorable prognosis, while patients with high levels of LDHA, TES and SIAH2 had poor survival outcomes (Figure 5B). Furthermore, the qPCR results indicated the mRNA expression levels of JMJD6, ANKZF1, CITED2, and ENO3 was obviously decreased in pancreatic cancer cells versus the normal pancreatic ductal epithelial cells, whereas those of LDHA, TES, and SIAH2 were oppisite (Figure 6).

### Evaluation of prognostic factors in PAAD

**FIGURE 7:**
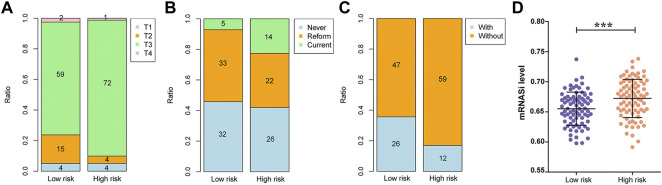
Associations between the risk score and clinical data as well as mRNAsi values. **(A)** The association between the risk score and pathologic T grading. **(B)** The association between the risk score and tobacco history. **(C)** The association between the risk score and radiotherapy. **(D)** The association between the risk score and mRNAsi values.

**FIGURE 8:**
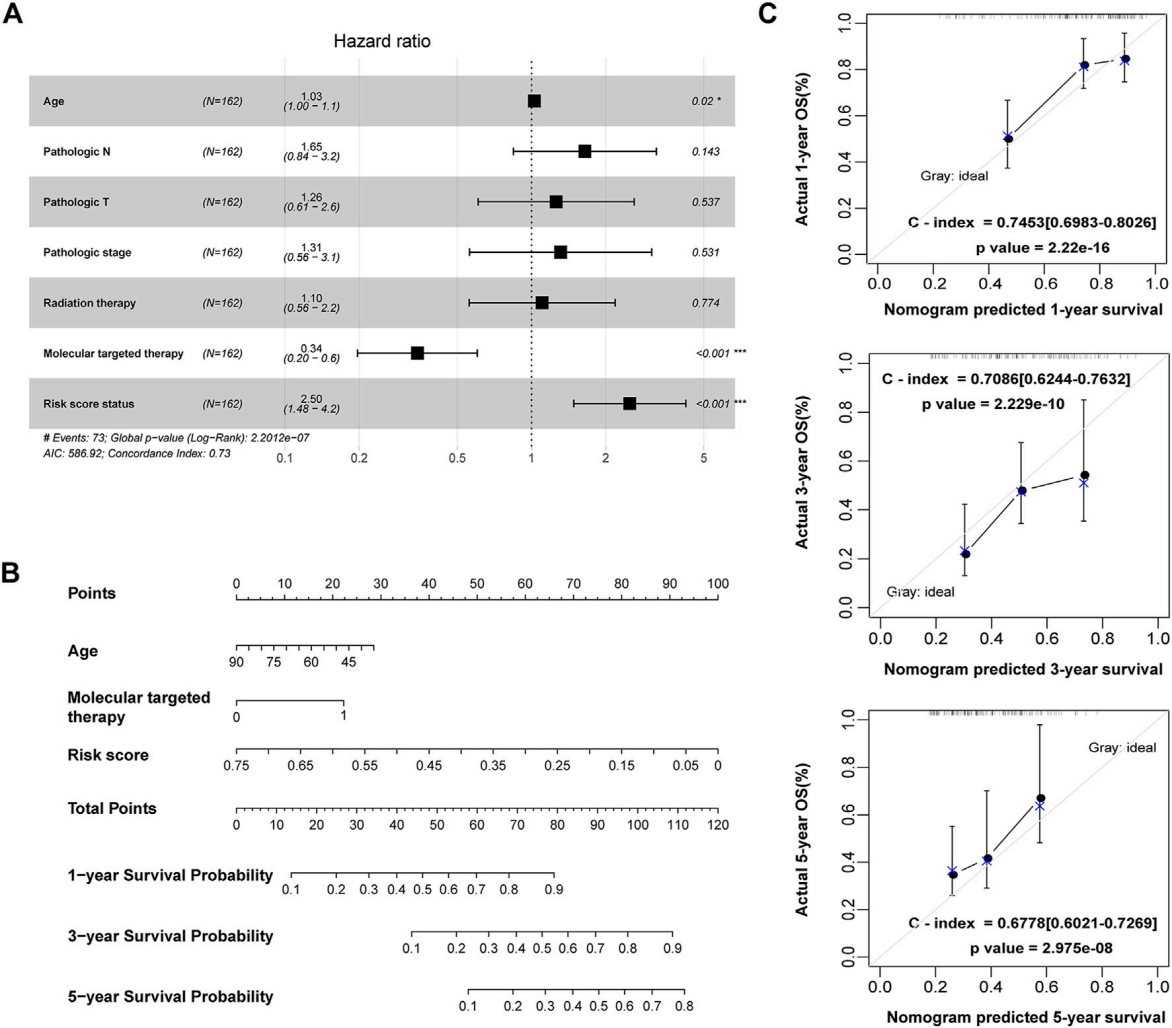
Construction of a nomogram for OS prediction in the TCGA PAAD dataset. **(A)** Forest plot of the multivariate Cox regression analysis of the risk score and clinicopathological parameters in PAAD. *p < 0.05, ***p < 0.001. **(B)** The nomogram incorporating risk score and clinical factors for survival prediction in PAAD. **(C)** The calibration curve of the nomogram for predicting the 1-, 3- and 5-year OS rates of PAAD patients in the training cohort. The *X*-axis represents the predicted OS rates, and the *Y*-axis represents the actual OS rates. The dashed line at 45° indicates the ideal performance, and the C-index was calculated to reflect the predictive accuracy of the nomogram.

Among the clinicopathological characteristics of 162 PAAD patients acquired from the TCGA dataset, three clinical parameters (pathologic T stage, smoking history and radiotherapy) were observed to be strongly associated with high risk scores (Table 1), and the distribution proportion of the three factors as well as mRNAsi variables between different risk groups was visualized (Figure 7). Univariate and multivariate Cox regression analyses revealed that age and targeted molecular therapy were significantly associated with OS. Moreover, multivariate analysis suggested that the eight-gene risk model was an independent prognostic factor for PAAD (HR = 2.503, *p* < 0.001) (Figure 8A and Table 2). In addition, the HR value of the risk score was higher than that of age and targeted molecular therapy, indicating that the risk score was more valuable for survival prediction in PAAD.

**TABLE 1:** Clinical characteristics and pathological parameters of PAAD patients.

Characteristics	N of case 162	Risk group		*p* Value
		Low risk (N = 81)	High risk (N = 81)	
Age (years)				
≤60	54	23	31	0.2432
>60	108	58	50	
Gender			
Male	89	45	44	0.1054
Female	100	63	37	
Pathologic M			
M0	74	33	41	0.9990
M1	3	1	2	
Pathologic N			
N0	44	25	19	0.2883
N1	114	53	61	
Pathologic T			
T1	8	4	4	0.0289
T2	19	15	4	
T3	131	59	72	
T4	3	2	1	
Pathologic stage			
Stage I	20	14	6	0.1247
Stage II	134	62	72	
Stage III	4	3	1	
Stage IV	3	1	2	
Chronic pancreatitis history			
Yes	13	4	9	0.1470
No	115	62	53	
Diabetes history			
Yes	36	21	15	0.4361
No	97	48	49	
Alcohol history			
Yes	95	52	43	0.1799
No	56	24	32	
Tobacco history			
Never	58	32	26	0.0371
Reform	55	33	22	
Current	19	5	14	
Ratiotherapy			
Yes	38	26	12	0.0138
No	106	47	59	
Recurrence			
Yes	101	52	49	0.9990
No	48	24	24	

**TABLE 2:** Univariate and multivariate Cox regression analysis of clinical factors.

Variables	Univariate analysis			Multivariate analysis		
	HR	95% CI	*p* Value	HR	95% CI	*p* Value
Age (mean ± SD)	1.030	1.008–1.053	**0.0053**	1.028	1.004–1.052	**0.0203**
Gender (Male/Female)	0.912	0.591–1.408	0.678	—	—	—
Pathologic_M (M0/M1)	1.83	0.434–7.719	0.403	—	—	—
Pathologic_N (N0/N1)	2.078	1.210–3.567	**0.0048**	1.646	0.844–3.210	0.1430
Pathologic_T (T1/T2/T3/T4)	1.713	1.087–2.700	**0.0116**	1.258	0.607–2.608	0.5370
Pathologic_stage (I/II/III/IV)	1.561	1.033–2.355	**0.0430**	1.313	0.561–3.072	0.5310
Chronic pancreatitis history (Yes/No)	1.091	0.518–2.295	0.8200	—	—	—
Diabetes history (Yes/No)	0.899	0.506–1.600	0.7160	—	—	—
Alcohol history (Yes/No)	1.103	0.689–1.765	0.6830	—	—	—
Tobacco history (Never/Reform/Current)	1.191	0.835–1.695	0.3360	—	—	—
Tumor recurrence (Yes/No)	1.613	0.970–2.683	0.0707	—	—	—
Radiation therapy (Yes/No)	0.509	0.284–0.910	**0.0151**	1.105	0.559–2.184	0.7740
Targeted molecular therapy (Yes/No)	0.489	0.310–0.772	**0.0017**	0.344	0.197–0.602	**0.0002**
RS prediction model (High/Low)	2.508	1.575–3.992	<**0.0001**	2.503	1.483–4.226	<**0.0001**

### Construction and evaluation of a predictive nomogram for PAAD prognosis

The 162 PAAD patients with complete clinical information in the TCGA dataset were used to construct the prognostic nomogram. By integrating the risk score and traditional prognostic factors, including age and targeted molecular therapy, we established a prognostic nomogram to predict the 1-, 3-, and 5-year OS of PAAD patients (Figure 8B). The C-indexes of the nomogram for 1-, 3-, and 5-year OS were 0.745 (95% CI: 0.698–0.803), 0.709 (95% CI: 0.624–0.763) and 0.678 (95% CI: 0.602–0.727), respectively (Figure 8C), suggesting that the 1-year, 3-year and 5-year survival rates predicted by the nomogram were close to the actual survival rates, and the nomogram showed superiority in 1- and 3-year survival prediction but not in 5-year survival prediction for PAAD patients.

### GSVA analysis between the low- and high-risk groups

**FIGURE 9:**
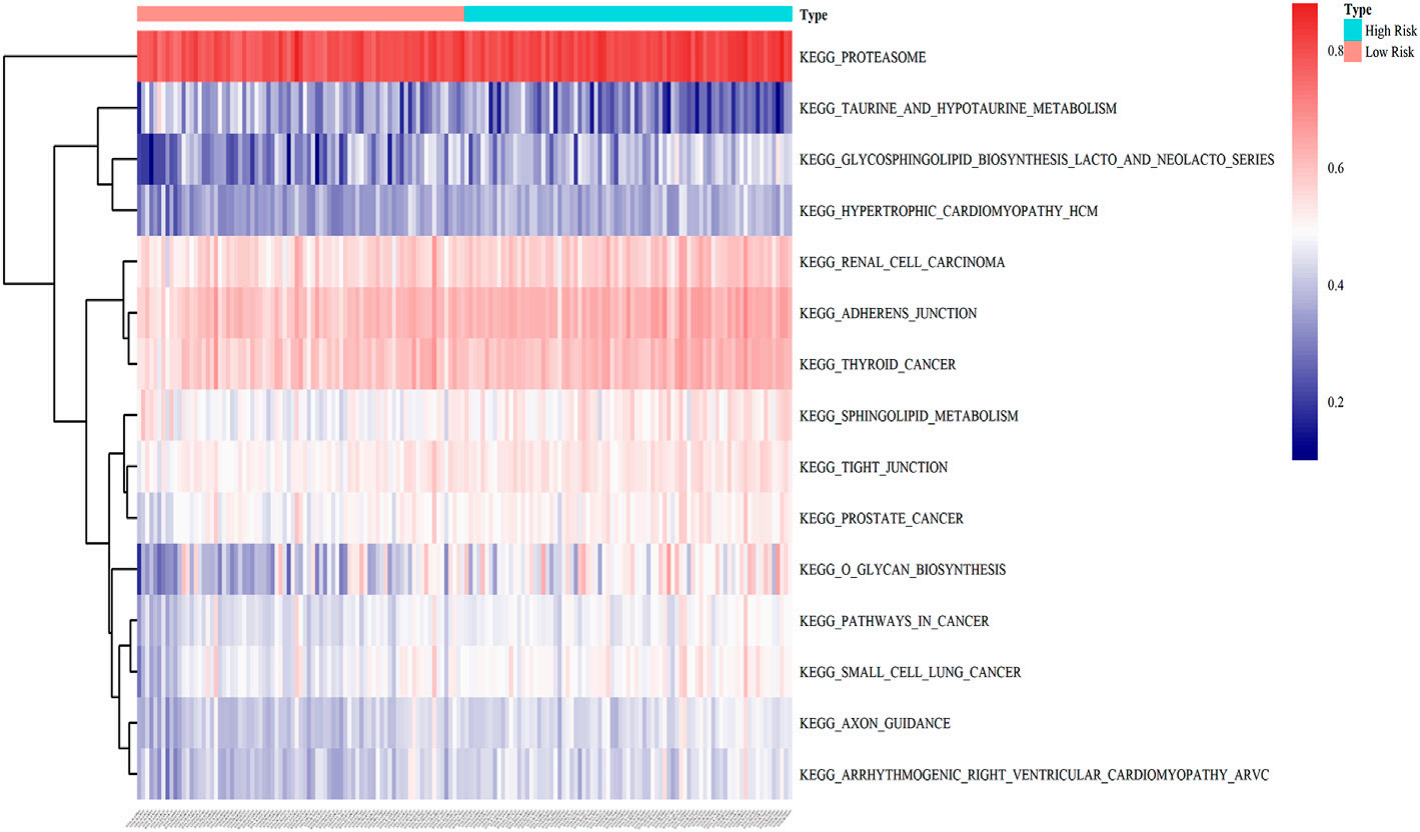
GSVA enrichment analysis of biological behaviors between the low- and high-risk groups in the training dataset. The heatmap was applied to visualize the top 15 distinct KEGG pathways arranged from small to large according to the *p* value; red indicates activated pathways, and blue indicates inhibited pathways.

To illustrate the potential molecular mechanisms of the eight-gene prognostic signature, 162 patients in the training cohort were classified into low- and high-risk groups. We further performed GSVA to estimate the difference in the pathway activation state of PAAD samples between the low- and high-risk groups, and a heatmap was used to visualize the top 15 distinct KEGG pathways arranged in order of *p* value from small to large. We found that several activation pathways of oncogenesis were remarkably enriched in the high-risk group, such as adherens junction, tight junction, sphingolipid metabolism, and pathways in cancer (Figure 9), indicating that abnormal expression of signature genes in PAAD is closely related to cancer-related pathways.

### Correlation between the eight-gene signature and immune status

**FIGURE 10:**
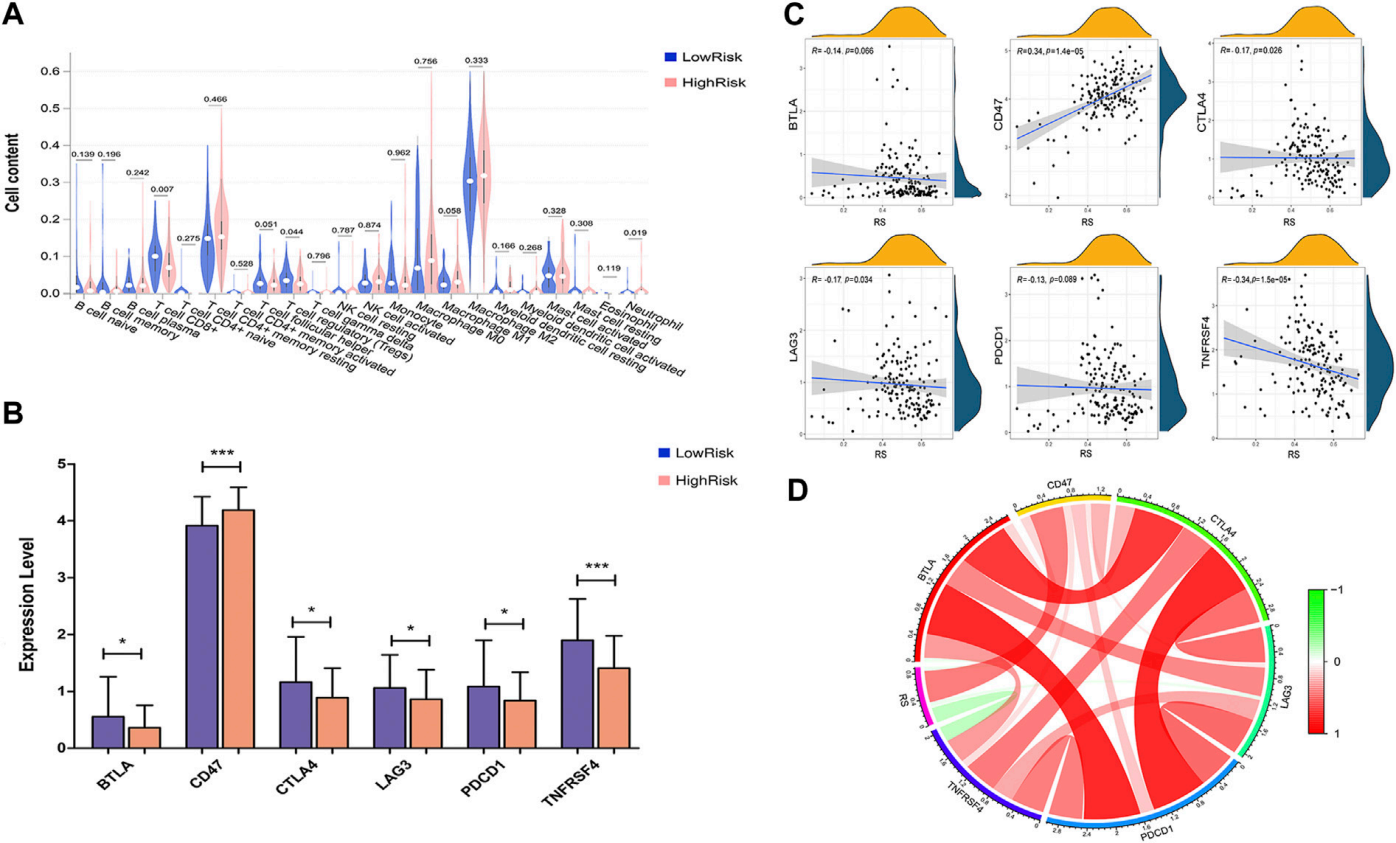
Correlation analysis between risk score and immune status. **(A)** The correlation between the risk score and infiltrating immune cells. **(B)** The correlation between risk score and immune checkpoint genes. **(C)** Correlation between risk score and immune checkpoint genes, including BTLA, CD274, CTLA4, LAG3, TNFRSF4 and PDCD1/PD-1. **(D)** Correlation analysis between risk score and the expression levels of immune checkpoint inhibitors. Red represents a positive correlation, and green represents a negative correlation.

To estimate the immunity relevance of our prognostic signature, the correlation of the risk score with infiltrating immune cells and immune checkpoint genes in PAAD tumor samples was analyzed. A remarkable difference in CD8^+^ T cells (*p* < 0.007), regulatory T cells (Tregs, *p* < 0.044) and neutrophils (*p* < 0.019) was found between the low- and high-risk groups (Figure 10A). In addition, the expression levels of immune checkpoint genes, including PDCD1/PD-1, CTLA4, LAG3, BTLA and TNFRSF4, were significantly decreased in the high-risk group, while the level of CD47 was dramatically increased in the high-risk group compared with the low-risk group (Figure 10B). Moreover, Pearson correlation analysis suggested that the prognostic risk signature was strongly inversely associated with CTLA4 (r = −0.17; *p* = 0.026), LAG3 (r = −0.17; *p* = 0.034) and TNFRSF4 (r = −0.34; *p* = 1.5e-05), whereas positively correlated with CD47 (r = 0.34; *p* = 1.4e-05; Figures 10C,D). These findings indicated that the eight-gene signature might play a critical role in regulating the immune response.

## Discussion

PAAD is one of the deadliest cancers in humans and is a hallmark of cellular and phenotypic heterogeneity (Melendez-Zajgla and Maldonado, 2021). The presence of CSCs may help explain the high mortality of pancreatic cancer, since the vital role of CSCs in the occurrence, development and recurrence of pancreatic cancer has been previously described in several studies (Hermann et al., 2007; Nassar and Blanpain, 2016; Stoica et al., 2020). As a key regulator of the cell response to hypoxia in the tumor microenvironment, HIF-1α is known to contribute to the maintenance of CSC stemness (Hao, 2015). Lv et al. (2019) reported that HIF-1*α* modulated the proliferation and differentiation of stem cells *via* the Wnt/*β*-catenin signaling pathway under hypoxic conditions, and activation of HIF-1*α* enhanced hypoxia-induced cancer stemness, thus facilitating cancer progression and resulting in unfavorable prognosis in PAAD patients (Colbert et al., 2015; Chen et al., 2019). To date, studies have clarified the prognostic value of gene signatures in the survival prediction of patients with pancreatic cancers, and prognostic signatures combined with conventional clinicopathological parameters such as TNM staging and tumor grade have been demonstrated to be superior to a single biomarker (Metzger et al., 2020; Abou Khouzam et al., 2021; Chen et al., 2021). However, few reports have well established reliable prognostic signatures for PAAD based on the combination of hypoxia and cancer stemness.

In this work, we identified a novel eight-gene signature for the survival prediction of PAAD patients based on comprehensive analyses of publicly available data. A prognostic risk model was established based on the eight-gene signature, in which NDST1, LDHA, TES and SIAH2 were significantly upregulated and correlated with poor prognosis, whereas the declined levels of JMJD6, ENO3, ANKZF1 and CITED was associated with adverse survival outcomes of PAAD patients. In addition, the expression levels of eight genes in pancreatic tumor cells was also verified by HPA database and qPCR analyses. Besides, the eight-gene signature was identified as an independent prognostic factor, and its excellent predictive efficiency was confirmed in both the training and external validation datasets. Furthermore, a prognostic nomogram incorporating the eight-gene signature and clinical factors was constructed and could reliably predict 1- and 3-year OS of PAAD patients. Overall, our findings highlighted the important clinical value of the eight-gene signature in the risk assessment and survival prediction of PAAD.

To better elucidate the molecular mechanisms underlying PAAD, we performed functional enrichment analysis of the identified HSRGs and found that they were closely correlated with angiogenesis, hypoxia response and the apoptotic process, and the HIF-1 signaling pathway and glycolysis/gluconeogenesis were the most enriched signaling pathways, suggesting that suggest these genes are tightly associated with cancer-related pathways. Additionally, GVSA analysis showed that adherens junction, tight junction and sphingolipid metabolism were abnormally developed in PAAD patients with high risk scores, revealing that the molecular alterations in the high-risk group were highly associated with the occurrence and development of PAAD. Collectively, these results provide important insights into the pathogenesis and development of PAAD.

Among the eight genes, the crucial roles of ENO3, LDHA and SIAH2 in the progression of pancreatic cancer have been demonstrated. ENO3, as a member of the human enolase (ENO) family catalyzing the transformation of 2-phosphoglycerate to phosphoenolpyruvate during glycolysis, was reported to inhibit the growth of cancer cells (Kong et al., 2016; Feng et al., 2021). Tan et al. (2020) reported that the expression level of ENO3 mRNA was remarkably downregulated in tumor tissues of pancreatic ductal adenocarcinoma (PDAC), and patients with decreased ENO3 levels had poor survival, suggesting that ENO3 is a promising biomarker for prognostic prediction in PADC patients. LDHA is an enzyme that promotes glycolytic processes by converting pyruvate to lactate, and its high expression is correlated with poor outcome in cancer patients (Brand et al., 2016). Knockdown of LDHA prevented tumor growth and metastasis by increasing the production of reactive oxygen species in several cancers (Rizwan et al., 2013; Jin et al., 2017). Previous studies demonstrated the strong correlation of LDHA overexpression with the poor prognosis of patients with pancreatic cancer, and the anticancer effect of LDHA inhibitors offered proof of LDHA as a potential therapeutic target for human cancers (Feng et al., 2018). SIAH2 functions as an E3 ubiquitin ligase that mediates the stabilization of HIF-1α and activates its downstream transcription, which reversely promotes SIAH2 expression through the PI3K/AKT pathway (Matsui-Hasumi et al., 2017). Inhibitors targeting SIAH2 and the SIAH2-HIF-1 axis altered the response of tumor cells to hypoxia and thus affected tumorigenesis and cancer progression (Xu and Li, 2021), highlighting the vital role of SIAH2 as a potential target for cancer therapy. Collectively, these results indicate that the stemness-related hypoxia genes ENO3, LDHA and SIAH2 are promising therapeutic targets in PAAD.

Nevertheless, the clinical significance of JMJD6, NDST1, TES, ANKZF1 and CITED2 has not been revealed in PAAD. Existing evidence suggests the crucial role of JMJD6 as a tumorigenic factor in several cancers, and the high expression of JMJD6 was found to promote the proliferation and survival of tumor cells and predicted the dismal prognosis of patients (Wang et al., 2014; Wong et al., 2019; Biswas et al., 2020). Previous studies have demonstrated the tumorigenic effects of NDST1 in primary glioblastoma and breast cancer, suggesting that it is a promising target for anticancer therapy (He et al., 2014; Xue et al., 2019). TES serves as a tumor suppressor in several cancers, such as gastric cancer and breast cancer, and overexpression of TES reduces the oncogenicity and metastasis of tumor cells (Okolicsanyi et al., 2014; Xue et al., 2019). Xu et al. (2020) proved the association of a glycolytic risk signature, including ANKZF1, with cancer progression and patient prognosis in renal cell carcinoma. Zhou et al. identified ANKZF1 as a prognostic biomarker in colon cancer, and overexpression of ANKZF1 could predict poor OS and recurrence-free survival (Zhou et al., 2019; Chen et al., 2020). CITED2 is highly expressed in many malignancies, including breast cancer, lung cancer, colon cancer and gastric cancer, and it plays critical roles in cancer metastasis and invasiveness (An et al., 2020). In addition, CITED2 is a direct product of HIF-mediated transcription and acts as a vital regulator of stem cell transcription factors in cancer stem-like cells (Rasti et al., 2018; Usui-Ouchi et al., 2020), suggesting that targeting CITED2 expression might be a novel therapeutic strategy for cancer treatment and relapse prevention. Therefore, the roles of JMJD6, NDST1, TES, ANKZF1 and CITED2 in PAAD should be further investigated.

The tumor microenvironment is an immune-related complex environment conducive to tumor cell survival and development. Existing research recognizes the significant effect of immune infiltration on tumorigenesis and metastasis and its association with patient survival in pancreatic cancer (Zhou et al., 2021). Activation of CD8^+^ T cells is crucial for the prevention of tumorigenesis and the induction of tumor regression and correlates with the long survival of patients with pancreatic cancer (Zhang et al., 2020). The prevalence of Treg cells showed a close relationship with tumor metastasis and poor survival of PDAC patients (Tang et al., 2014). Notably, Mota Reyes et al. (2020) revealed that depletion of Tregs could alter the TIME and accelerate pancreatic carcinogenesis and disease progression partially by inducing pathological CD4^+^ T-cell responses. By analyzing the infiltration of immune cells in PAAD samples, we found a remarkable decrease in CD8^+^ T cells (*p* < 0.007) and Treg cells (*p* < 0.044) in the high-risk group, which may partly explain the poor prognosis in the high-risk group from an immunological perspective. CTLA-4 and PD-1 are the most studied coinhibitory receptors of T cell receptor signaling, and blockade of CTLA-4 and PD-1 results in T-cell activation and enhanced immune responses (Lee et al., 2018; Henriksen et al., 2019). A preclinical study proved that a CTLA-4 antagonist combined with gemcitabine was safe and tolerated in patients with metastatic PDAC (Aglietta et al., 2014), and PD-1 inhibitors along with chemotherapy showed antitumor effects on advanced PDAC patients, although single-agent checkpoint inhibitors showed disappointing limited activity in pancreatic cancer (Weiss et al., 2017). We further analyzed the association between the risk score and immune checkpoint genes and found that patients in the low-risk group showed higher levels of CTLA4, PD-1, BTLA, LAG3 and TNFRSF4, suggesting that these patients might be more likely to benefit from the combined use of immune checkpoint inhibitors. Collectively, our findings shed light on the close correlation between the risk score and immune status in PAAD.

Our study comprehensively analyzed and identified the HSRGs associated with prognosis on the basis of public TCGA-PAAD database. More importantly, we developed a eight-gene prognostic signature with good capacity in predicting the survival outcomes of PAAD patients. However, our research presents several limitations. The construction and verification of the nomogram for predicting the OS of PAAD patients were conducted based on the TCGA dataset, and external dataset validation needs to be performed in the future. Additionally, we validated the gene expression of the eight signature genes at the cellular level, their expression in PAAD patient specimens also needs further experimental verification. Moreover, more researches are required to elucidate the underlying molecular mechanisms of the eight genes in the pathogenesis and progression of PAAD.

## Conclusion

In summary, we developed a novel eight-gene signature and a prognostic nomogram that could reliably predict patient survival in PAAD. Our prognostic gene signature could be beneficial for the risk stratification and prognostic prediction of PAAD.

## Data Availability

The datasets presented in this study can be found in online repositories. The names of the repository/repositories and accession number(s) can be found in the article/Supplementary Material.

## Supplementary material

Supplementary Figure S1 Top 3 hypoxia genes correlated with mRNAsi. Green represents the top 3 genes negatively correlated with mRNAsi, while red represents the top 3 genes positively correlated with mRNAsi.

Supplementary Figure S2 Identification of the optimal genes used to develop the prognostic signature by the LASSO regression algorithm. **(A)** Selection of the optimal parameter (lambda) in the LASSO model. The two vertical lines are lambda.min and lambda.lse. **(B)** LASSO coefficient profiles of eight OS-related genes with nonzero coefficients determined by the optimal lambda.

Supplementary Table S2 The 108 hypoxia genes significantly associated with mRNAsi.

Supplementary Table S3 The 45 HSRGs strongly correlated with OS of PAAD patients.
