# Supplementary material for: Development and validation of a hypoxia-stemness-based prognostic signature in pancreatic adenocarcinoma
Source: Front Pharmacol. 2022 Jul 21;13:939542. doi: 10.3389/fphar.2022.939542 (PMC9350896; doi:10.3389/fphar.2022.939542)
Supplement: Supplementary file 3 [file Table1.DOCX]

Supplemental Table 1. The primer sequences of the eight signature genes and GAPDH

| **Gene** | **Primer sequences** |
| --- | --- |
| ANKZF1 | F: ATGCTCCGGTCTTTCAGGG  R: GGTCTGGTCACAAGTTGAACAAA |
| CITED2 | F: CCTAATGGGCGAGCACATACA  R: GGGGTAGGGGTGATGGTTGA |
| ENO3 | F: GGCTGGTTACCCAGACAAGG  R: TCGTACTTCCCATTGCGATAGAA |
| JMJD6 | F: TTGGACCCGGCACAACTACTA R: TCTGCCCTTTCCACGTTATCC |
| LDHA | F: ATGGCAACTCTAAAGGATCAGC  R: CCAACCCCAACAACTGTAATCT |
| NDST1 | F: CTGCCTGTTCAGCGTTTTCAT R: CGAGTAGAGGCTCTCCACAAA |
| SIAH2 | F: TCTTCGAGTGTCCGGTCTG  R: CGGCATTGGTTACACACCAG |
| TES | F: ATGGGCTTAGGTCACGAGC R: TCCCACTTTTCGATCCTCTTCA |
| GAPDH | F: CTGGGCTACACTGAGCACC  R: AAGTGGTCGTTGAGGGCAATG |
