# Supplementary figures and images for: Development and validation of a hypoxia-stemness-based prognostic signature in pancreatic adenocarcinoma
Source: Front Pharmacol. 2022 Jul 21;13:939542. doi: 10.3389/fphar.2022.939542 (PMC9350896; doi:10.3389/fphar.2022.939542)

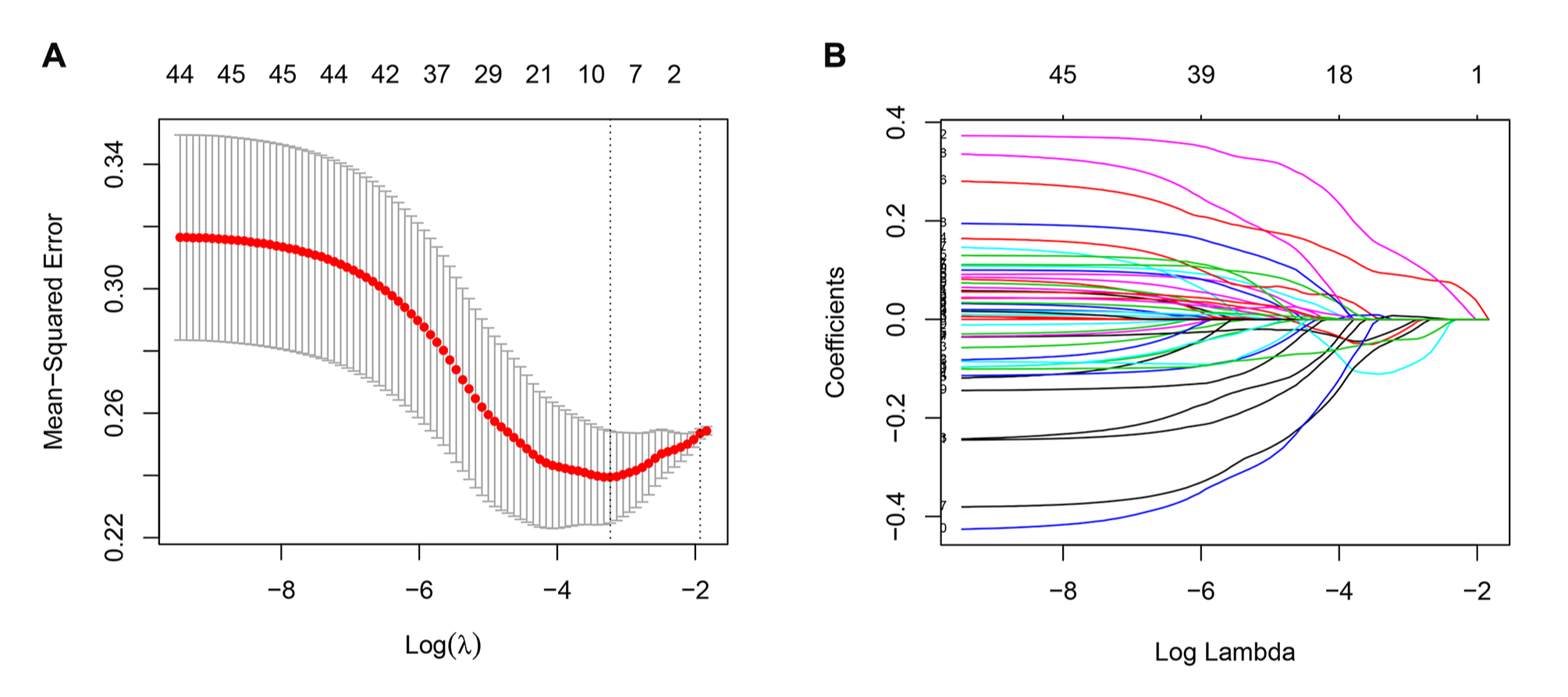

Supplement: Supplementary file 4 [file Image2.TIF]

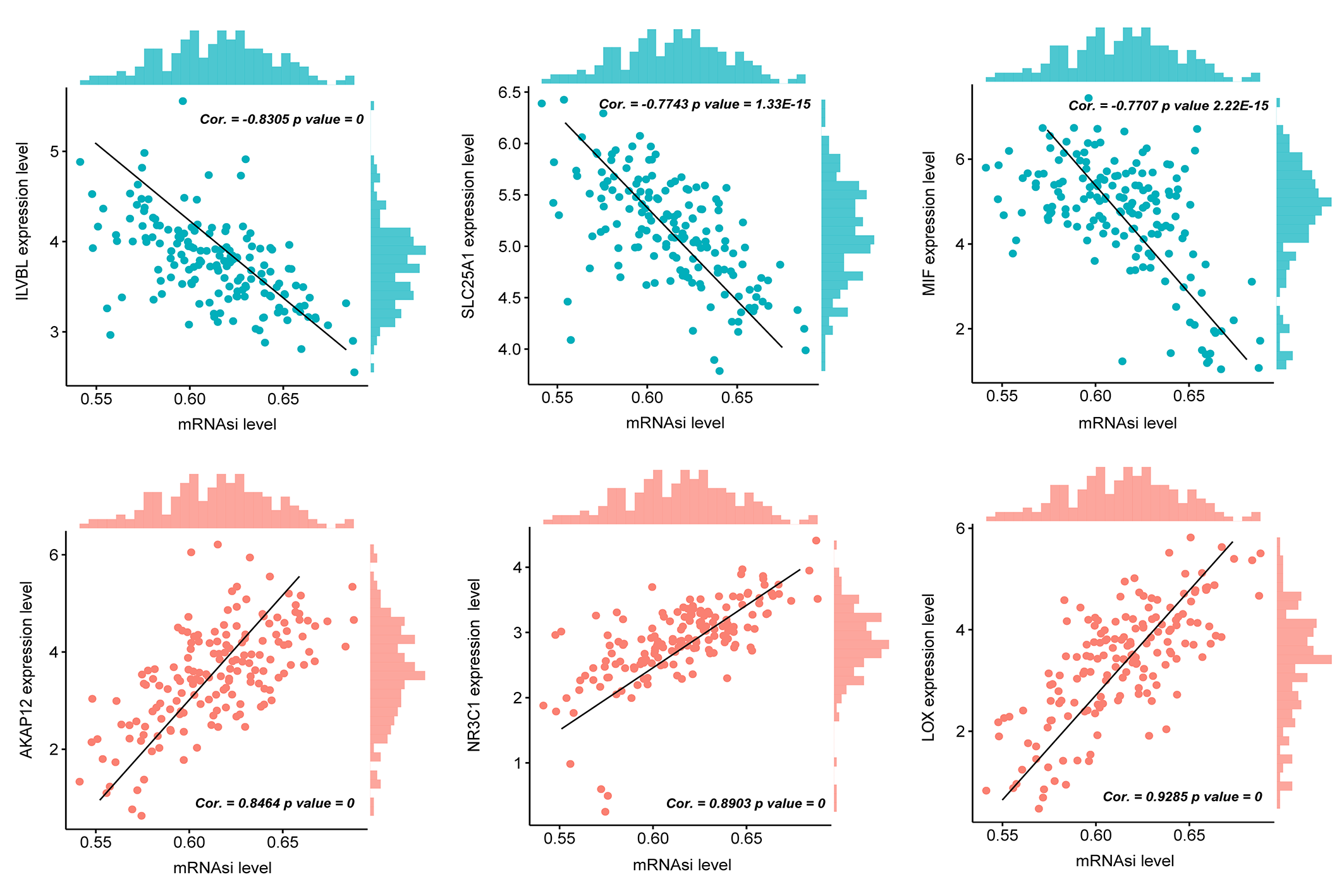

Supplement: Supplementary file 5 [file Image1.TIF]
